# Exposure to *Leishmania* spp. and sand flies in domestic animals in northwestern Ethiopia

**DOI:** 10.1186/s13071-015-0976-1

**Published:** 2015-07-08

**Authors:** Iva Rohousova, Dalit Talmi-Frank, Tatiana Kostalova, Nikola Polanska, Tereza Lestinova, Aysheshm Kassahun, Daniel Yasur-Landau, Carla Maia, Roni King, Jan Votypka, Charles L. Jaffe, Alon Warburg, Asrat Hailu, Petr Volf, Gad Baneth

**Affiliations:** Department of Parasitology, Faculty of Science, Charles University in Prague, Vinicna 7, 128 44 Prague 2, Czech Republic; School of Veterinary Medicine, The Hebrew University of Jerusalem, P.O. Box 12, Rehovot, 76100 Israel; Medical Parasitology Unit, Global Health and Tropical Medicine, Institute of Hygiene and Tropical Medicine, Universidade Nova de Lisboa, Rua da Junqueira 100, 1349-008 Lisboa, Portugal; Israel Nature and Parks Authority, 3 Am Ve’Olamo Street, Jerusalem, 95463 Israel; Department of Microbiology and Molecular Genetics, The Institute for Medical Research Israel-Canada, The Kuvin Centre for the Study of Infectious and Tropical Diseases, The Hebrew University - Hadassah Medical School, The Hebrew University of Jerusalem, Jerusalem, 91120 Israel; Department of Microbiology, Immunology and Parasitology, Faculty of Medicine, Addis Ababa University, P.O. Box 9086, Addis Ababa, Ethiopia

**Keywords:** Visceral leishmaniasis, Ethiopia, Domestic animals, Serology, PCR, *Phlebotomus orientalis*, *Leishmania donovani*, Sand fly saliva

## Abstract

**Background:**

Human visceral leishmaniasis caused by *Leishmania donovani* is considered an anthroponosis; however, *Leishmania*-infected animals have been increasingly reported in *L. donovani* foci, and the role of these animals as reservoirs for human *L. donovani* infection remains unclear.

**Methods:**

We conducted a study of domestic animals (goats, sheep, cows, dogs, and donkeys) in three *L. donovani* foci in northwestern Ethiopia. Domestic animals were screened for *Leishmania* DNA and for anti-*L. donovani* IgG. Serum anti-sand fly saliva antibodies were used as a marker of exposure to the vector sand fly, *Phlebotomus orientalis*.

**Results:**

Of 546 animals tested, 32 (5.9 %) were positive for *Leishmania* DNA, with positive animals identified among all species studied. Sequencing indicated that the animals were infected with parasites of the *L. donovani* complex but could not distinguish between *L. infantum* and *L. donovani*. A total of 18.9 % of the animals were seropositive for anti-*L. donovani* IgG, and 23.1 % of the animals were seropositive for anti-*P. orientalis* saliva IgG, with the highest seroprevalence observed in dogs and sheep. A positive correlation was found between anti-*P. orientalis* saliva and anti-*L. donovani* IgGs in cows, goats, and sheep.

**Conclusions:**

The detection of *L. donovani* complex DNA in the blood of domestic animals, the reported seroprevalence to the *L. donovani* antigen, and the widespread exposure to sand fly saliva among domestic animals indicate that they are frequently exposed to *Leishmania* infection and are likely to participate in the epidemiology of *Leishmania* infection, either as potential blood sources for sand flies or possibly as parasite hosts.

**Electronic supplementary material:**

The online version of this article (doi:10.1186/s13071-015-0976-1) contains supplementary material, which is available to authorized users.

## Background

Leishmaniasis, a protozoan disease that is transmitted by sand flies (Diptera: Phlebotominae) and caused by parasites of the genus *Leishmania* (Kinetoplastida: Trypanosomatidae), is a neglected tropical and subtropical disease endemic to 98 countries worldwide. In East Africa, life-threatening human visceral leishmaniasis (VL) is caused by *Leishmania donovani* and primarily affects the poor due to the lack of preventive measures and reduced access to health care facilities [1].

The optimal strategy for controlling this disease depends on understanding the epidemiology of VL, including its local transmission cycles. Leishmaniasis caused by *L. donovani* is believed to be an anthroponosis. However, in Latin America and the Mediterranean Basin, the closely related species *L. infantum* causes a zoonosis for which canids are the main reservoirs [2]. Controlling zoonoses involving domestic or sylvatic transmission requires a more complex intervention than would be necessary if humans were the only hosts. Several *Leishmania*-infected animals have been previously reported in *L. donovani* foci, including wild and domestic animals [3–5]. However, the role of these animals as parasite hosts or, possibly, as reservoirs for human *L. donovani* VL remains unclear and requires further examination.

Our study focused on the detection of *Leishmania* infections in domestic animals in three VL foci in northwestern Ethiopia. Domestic animals were screened for *Leishmania* DNA and anti-*L. donovani* IgG in their peripheral blood to detect infection and exposure to *Leishmania*, respectively. Additionally, anti-sand fly saliva antibodies were used as a marker of exposure [6] to *Phlebotomus orientalis*, the suspected vector of *L. donovani* in northwestern Ethiopia [7, 8]. The findings from this study could be used to further study the involvement of domestic animals in the transmission cycle of VL.

## Methods

### Study sites and sample collection

Animal blood and serum samples were collected in Addis Zemen, Humera, and Sheraro, three localities in northwestern Ethiopia endemic to human VL. In the Humera district (Tigray region), several outbreaks of VL have been recorded since 1970. Addis Zemen (Amhara region) and Sheraro (Tigray region) are sustained VL foci characterized by a local transmission cycle supported by migrant agricultural laborers returning from Humera [1].

Animal surveys were conducted during two field studies. In October 2010, 266 samples were collected in Addis Zemen and Sheraro, and in November 2010, an additional 280 samples were obtained in Humera (Table 1). For DNA extraction, samples of whole blood (with anticoagulant) were transported to the Hebrew University of Jerusalem (Israel), where extraction was performed. For serological testing, serum samples treated with a 1 % azide solution were transported to Charles University in Prague (the Czech Republic) and stored at −70 °C.

**Table 1:** Serum samples collected from October to November 2010 in Ethiopian VL foci

	Addis Zemen	Sheraro	Humera	Total
Cow	62	26	16	104
Dog	19	7	8	34
Donkey	3	11	6	20
Goat	0	106	133	239
Sheep	27	5	117	149
Total	111	155	280	546

### Ethical approval

The study was approved by the Ethiopian National Research Ethics Review Committee (NRERC), under approval no. 3.10/3398/04. Consent was obtained from the owners of the domestic animals for the collection of blood samples by a veterinarian. International animal experimentation guidelines were followed.

### DNA extraction and PCR amplification

DNA was extracted from whole blood using the guanidine thiocyanate technique [9]. DNA was tested for *Leishmania* spp*.* infection via kDNA real-time PCR as previously described [10, 11]. Samples that tested positive were further tested by *Leishmania* internal transcribed spacer 1 (ITS1) real-time PCR and high-resolution melt analysis (ITS1-HRM PCR) [12]. Samples that tested positive by ITS1-HRM PCR were further assessed via conventional PCR to amplify a larger segment of ITS1 [13]. All samples were tested in duplicate, and the results were compared with positive controls: *L. infantum* (MCAN/IL/2002/Skoshi), *L. tropica* (MHOM/IL/2005/ LRC-L1239), and *L. major* (MHOM/TM/1973/5ASKH) promastigotes. The negative controls included blood samples obtained from five Israeli dogs that had tested negative for *Leishmania* by PCR. All positive PCR products were submitted for DNA sequencing to the Center for Genomic Technologies at the Hebrew University of Jerusalem. The derived DNA sequences were compared with sequences in GenBank using the NCBI BLAST program (www.ncbi.nlm.nih.gov/BLAST). The percentage of positive animals for each species was calculated based on positive kDNA PCR results followed by sequencing. Samples were considered positive for *Leishmania* only if their kDNA sequence demonstrated the closest BLAST match to *Leishmania* and was at least 80 % identical. A species was considered to be identified only when its ITS1 sequence shared 99 to 100 % identity with an existing GenBank sequence.

### Discrimination between *Leishmania infantum* and *Leishmania donovani*

As ITS1-HRM PCR does not discriminate between *L. infantum* and *L. donovani* infections [12], samples that tested positive for the *L. donovani* complex were further evaluated using conventional PCR to determine the species. Two independent PCR assays were carried out to amplify fragments of the *Leishmania* cysteine protease B (CPB) gene [14, 15]. Furthermore, amplification of the heat shock protein 70 (HSP70) gene, followed by restriction fragment length polymorphism analysis was also attempted for species discrimination [16]. The same positive and negative controls used for ITS1-HRM PCR were employed.

A phylogenetic analysis was carried out using Kalign (www.ebi.ac.uk/tools/msa/kalign/) and BioEdit softwares. Only well-defined ITS sequences that were unambiguously assigned to the species *L. donovani* or *L. infantum* were downloaded from the GenBank database and used in the analysis (Additional file 1). The final alignment included 286 characters and is available upon request. Phylogenetic analyses of the ITS datasets were performed with PhyML for maximum likelihood (ML); the best-fitting model [GTR + *I* + Γ] of sequence evolution was assessed using Modeltest 3.7 software and bootstrapped with 1000 replicates.

### Anti-*Leishmania donovani* IgG antibodies

An ELISA was used to measure specific anti-*L. donovani* IgG. Wells (CovaLink NH, Nunc) were coated with *L. donovani* promastigotes (Ethiopian strain MHOM/ET/67/HU3, 10^5^ cells per well) in 20 mM carbonate-bicarbonate buffer (pH 9.25) overnight at 4 °C and incubated with 6 % blocking solution for 60 min at 37 °C. Serum samples were diluted in 2 % blocking solution and incubated in duplicate for 60 min at 37 °C. Thereafter, peroxidase-conjugated secondary antibodies were added, followed by 45 min of incubation at 37 °C. For details on the blocking solutions, sample dilutions, and conjugates employed in these assays, see Additional file 2. Absorbance was measured using a Tecan Infinite M200 microplate reader (Schoeller) at 492 nm.

Hyperimmune sera from laboratory-bred mice experimentally infected with *L. donovani* served as positive controls. Negative serum samples were obtained from healthy cattle (n = 33), horses (as controls for the donkeys; n = 9), goats (n = 21), and sheep (n = 32) from the Czech Republic, which is a sand fly- and *Leishmania*-free country. Canine-negative (n = 15) and canine-positive (n = 2) control sera were obtained during a previous study [17] from laboratory-bred beagles with no history of exposure to sand flies or *Leishmania* or from *Leishmania*-positive dogs, respectively.

### Anti-sand fly saliva IgG antibodies

To estimate the exposure of domestic animals to *P. orientalis*, anti-saliva IgG antibodies were measured via ELISA. The same protocol applied for anti-*Leishmania donovani* IgG was used, with the following modifications: wells were coated with a salivary gland homogenate (corresponding to 0.2 gland/well, prepared as previously described [18]), and serum samples were incubated in duplicate for 90 min at 37 °C. Hyperimmune sera from laboratory-bred mice exposed solely to *P. orientalis* served as a positive control. The same negative controls employed for the anti-*L. donovani* ELISA were also used here.

To assess the possible cross-reactivity of *P. orientalis* salivary gland homogenate with IgG antibodies against the saliva of other sand fly species, sera from mice and dogs that were experimentally exposed to a single sand fly species were used. Canine sera positive for anti-*P. perniciosus* and anti-*L. longipalpis* IgG antibodies were available from previous experiments in laboratory-bred beagles exposed solely to *P. perniciosus* [17] and *L. longipalpis* [18], respectively, the two proven vectors of *L. infantum*. The ELISA protocol described in Additional file 2 was applied with one modification: the sera were diluted 1:500. For the murine sera, the applied ELISA protocol was modified as follows: low-fat, dry milk (Bio-Rad) was used as a blocking solution and diluent for the serum samples (1:200), and goat anti mouse IgG:HRP (AbD SEROTEC, STAR120P) diluted 1:1000, was used as a secondary antibody. The serum samples were obtained from BALB/c mice subjected to more than ten repeated exposures solely to *P. orientalis* (Ethiopia), *P. papatasi* (Turkey), *P. duboscqi* (Senegal), *P. arabicus* (Israel), or *Sergentomyia schwetzi* (Ethiopia). The experiments were approved by the Committee on the Ethics of Animal Experiments of Charles University in Prague (Permit Number: 24773/2008-10001) and were performed under a Certificate of Competency (Registration Number: CZU 934/05), in accordance with an Examination Order approved by the Central Commission for Animal Welfare of the Czech Republic.

### Statistical analysis

For seroprevalence, cut-off values were calculated by the addition of three standard deviations to the mean optical density (OD) of the control sera. The differences in antibody levels between localities were analyzed using the nonparametric Wilcoxon Rank-Sum Test for Differences in Medians. Spearman’s rank correlation matrix was used to assess the correlation between the variables. Statistical analyses were performed using NCSS 6.0.21 software, and the p-value was set at 0.05.

## Results

### Prevalence of *Leishmania* infection

The overall prevalence of *Leishmania* DNA detected via PCR was 5.9 % (32/546) (Table 2, Additional file 3). None of the 546 tested domestic animals presented visible clinical signs associated with leishmaniasis. Of the 32 animals that tested positive by kDNA PCR, nine were also positive for ITS1 PCR (Table 2, Additional file 3). The majority of *Leishmania*-positive animals (30 out of 32) were found in Humera, with the highest prevalence observed in cows (18.8 %). At the other localities, only one donkey in Sheraro and one dog in Addis Zemen were found to be positive for *Leishmania* (Table 2, Additional file 3).

**Table 2:** *Leishmania* PCR positivity in samples from Ethiopian animals

	*Leishmania* kDNA positive/total animals sampled (% positive)				*Leishmania* ITS1 positive (% positive)
Species	Addis Zemen	Sheraro	Humera	Total	Total
Cow	0/62	0/26	3/16 (18.8 %)	3/104 (2.9 %)	1 (1 %)
Dog	1/19 (5.3 %)	0/7	1/8 (12.5 %)	2/34 (5.9 %)	1 (2.9 %)
Donkey	0/3	1/11 (9.1 %)	1/6 (16.7 %)	2/20 (10.0 %)	0
Goat	0/0	0/106	16/133 (12.0 %)	16/239 (6.7 %)	3 (1.3 %)
Sheep	0/27	0/5	9/117 (7.7 %)	9/149 (6.0 %)	4 (2.7 %)
Total	1/111 (0.9 %)	1/155 (0.6 %)	30/280 (10.7 %)	32/546 (5.9 %)	9 (1.6 %)

**Fig. 1:**
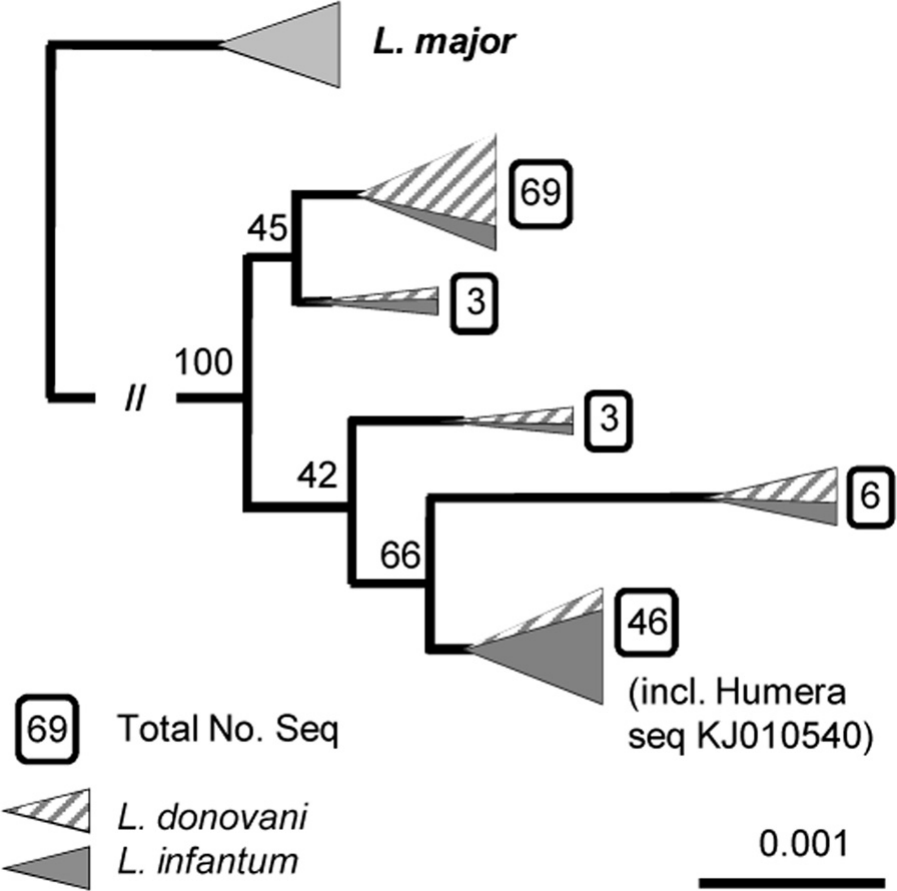
Phylogenetic analysis of the *Leishmania* ITS1 sequence in a sheep from Humera, Ethiopia. Maximum likelihood (ML) phylogenetic analysis of a 286 bp *Leishmania* sequence amplified from the blood of a sheep from Humera, Ethiopia [GenBank:KJ010540]. Only well-defined ITS sequences that could be unambiguously assigned to a species (*L. donovani* or *L. infantum*) were downloaded from the GenBank database and used for the following analysis (Additional file 1). The ITS sequences were aligned using Kalign (www.ebi.ac.uk/Tools/msa/kalign/), and the resulting alignments were edited manually using the BioEdit software program to remove unambiguous positions. The final alignment included 286 characters and is available upon request. Phylogenetic analyses of the ITS datasets were performed with PhyML for ML (the best-fitting model [GTR + *I* + Γ] of sequence evolution was assessed using Modeltest 3.7 and bootstrapped with 1000 replicates; likelihood: loglk = −404.139)

A total of nine ITS1 DNA sequences, 265 bp long and 99 % identical to *L. infantum*/*L. donovani* sequences, were obtained via ITS1-HRM-PCR. None of the animal samples yielded positive PCR results when targeting the CPB and HSP70 genes. A DNA sequence was obtained for only a single longer ITS1 amplicon from one sheep originating in Humera. This sequence (314 bp, [GenBank:KJ010540]) shares 100 % identity with sequences from both *L. infantum* and *L. donovani* with 100 % coverage, and its phylogeny did not permit discrimination between these two closely related species (Fig. 1).

### Anti-*Leishmania donovani* IgG antibodies

**Fig. 2:**
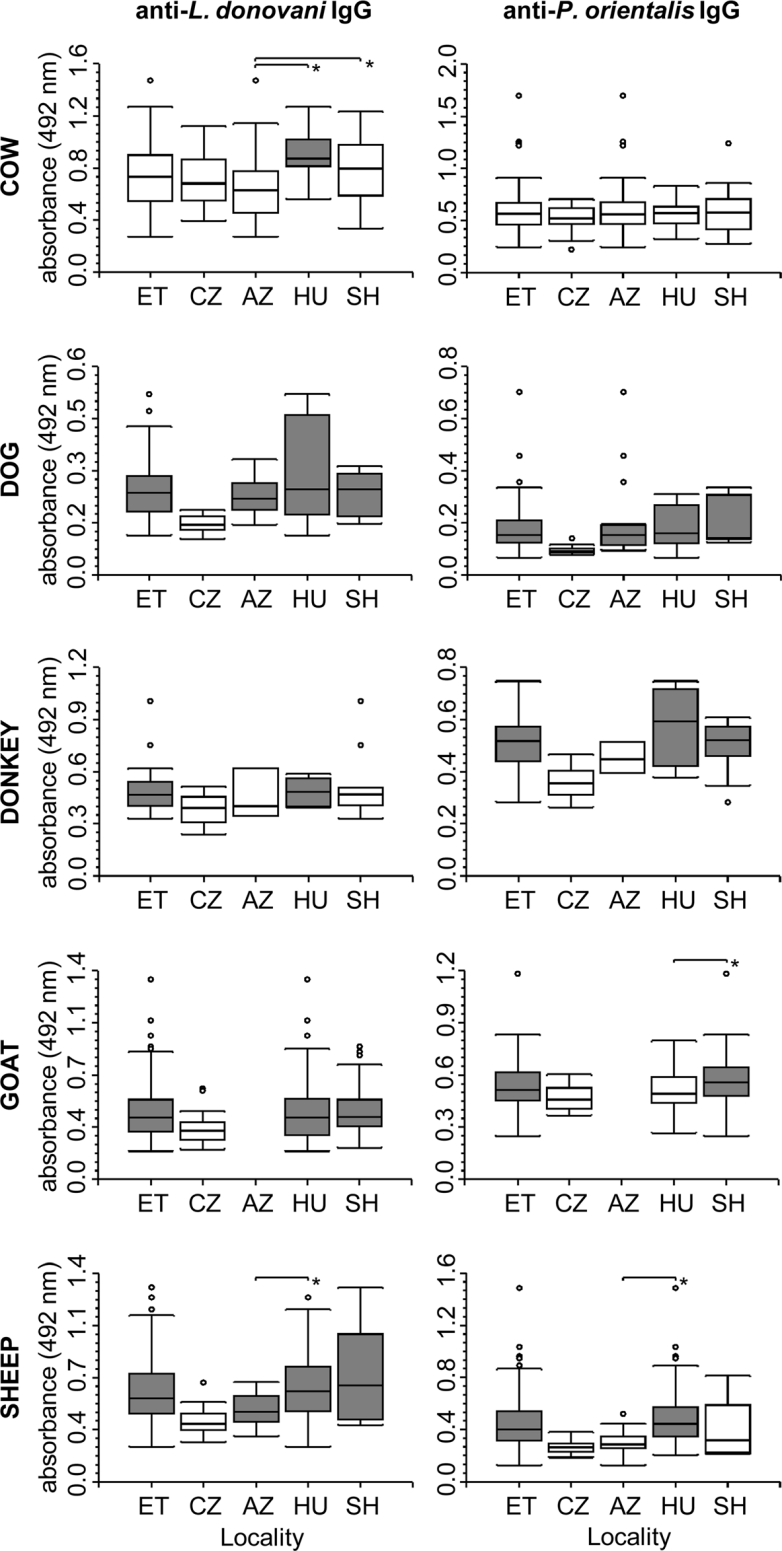
Serological survey of domestic animals in Ethiopia. IgG antibodies against *Leishmania donovani* promastigotes or *Phlebotomus orientalis* saliva in all serum samples collected from domestic animals in Ethiopia (ET) from Addis Zemen (AZ), Humera (HU), and Sheraro (SH). Significant differences compared with the Czech controls (CZ) are highlighted in gray. The asterisk represents differences between the 3 localities (AZ, HU, and SH) in Ethiopia

Seropositive animals were found for every species tested. The overall seroprevalence of anti-*L. donovani* IgG in the Ethiopian samples was 18.9 % (103/546) (Table 3; Fig. 2). Across all localities tested, the highest seropositivity was observed in dogs (overall 55.9 %) and the lowest in cows and donkeys (Table 3). Of the 32 animals that tested positive for *Leishmania* DNA, 12 animals also demonstrated seropositivity for the *L. donovani* antigen: 1 donkey, 3 goats, and 8 sheep (Additional file 3).

**Table 3:** Seropositivity of Ethiopian animals for *Leishmania donovani* IgG. The cut-off value was calculated as the mean optical density in the control animals plus 3 standard deviations (details provided in the Methods)

		Anti-*L. donovani* IgG positive/total animals sampled (% seropositive)			
Species	Cut-off	Addis Zemen	Sheraro	Humera	Total
Cow	1.298	1/62 (1.6 %)	0/26 (0 %)	0/16 (0 %)	1/104 (1.0 %)
Dog	0.223	9/19 (47.4 %)	5/7 (71.4 %)	5/8 (62.5 %)	19/34 (55.9 %)
Donkey	0.652	0/3 (0 %)	2/11 (18.2 %)	0/6 (0 %)	2/20 (10.0 %)
Goat	0.675		10/106 (9.4 %)	15/133 (11.3 %)	25/239 (10.5 %)
Sheep	0.648	1/27 (3.7 %)	3/5 (60.0 %)	52/117 (44.4 %)	56/149 (37.6 %)
Total		11/111 (9.9 %)	20/155 (12.9 %)	72/280 (25.7 %)	103/546 (18.9 %)

Apart from the cows, all of the Ethiopian animal species exhibited significantly higher levels of anti-*L. donovani* IgG compared with control animals (Fig. 2). Geographically, significantly higher levels of anti-*L. donovani* IgG were observed in all animal species from Humera and in dogs, goats, and sheep from the other localities tested, when compared with control animals (Fig. 2).

### Anti-*Phlebotomus orientalis* saliva IgG antibodies

The seroprevalence of anti-*P. orientalis* IgG in Ethiopian animals was 23.1 % (126/546) (Table 4). Seropositive animals were identified for every species and at every locality tested. In Addis Zemen and Sheraro, the highest seroprevalence was observed in dogs (57.9 and 57.1 %, respectively), whereas in Humera, the highest seroprevalence was among donkeys, dogs, and sheep (66.7, 62.5, and 57.3 %, respectively) (Table 4).

**Table 4:** Seropositivity of Ethiopian animals for *Phlebotomus orientalis* saliva IgG. The cut-off value was calculated as the mean optical density in the control animals plus 3 standard deviations (details provided in the Methods)

		Anti-*P. orientalis* IgG positive/total animals sampled (% seropositive)			
Species	Cut-off	Addis Zemen	Sheraro	Humera	Total
Cow	0.876	4/62 (6.5 %)	1/26 (3.8 %)	0/16 (0 %)	5/104 (4.8 %)
Dog	0.143	11/19 (57.9 %)	4/7 (57.1 %)	5/8 (62.5 %)	20/34 (58.8 %)
Donkey	0.550	0/3 (0 %)	3/11 (27.3 %)	4/6 (66.7 %)	7/20 (35.0 %)
Goat	0.685		17/106 (16.0 %)	6/133 (4.5 %)	23/239 (9.6 %)
Sheep	0.410	3/27 (11.1 %)	1/5 (20.0 %)	67/117 (57.3 %)	71/149 (47.7 %)
Total		18/111 (16.2 %)	26/155 (16.8 %)	82/280 (29.3 %)	126/546 (23.1 %)

Apart from cows, all of the animal species from Ethiopia exhibited significantly (p < 0.05) higher anti-*P. orientalis* IgG seroreactivity compared with control animals (Fig. 2). Geographically, elevated levels of anti-*P. orientalis* IgG were observed in dogs, donkeys, and sheep from Humera and in dogs, donkeys, and goats from Sheraro. In Addis Zemen, only dogs exhibited significantly higher seroreactivity than control animals. The seroreactivities in the bovine samples were similar to those in control animals, regardless of the locality (Fig. 2).

**Fig. 3:**
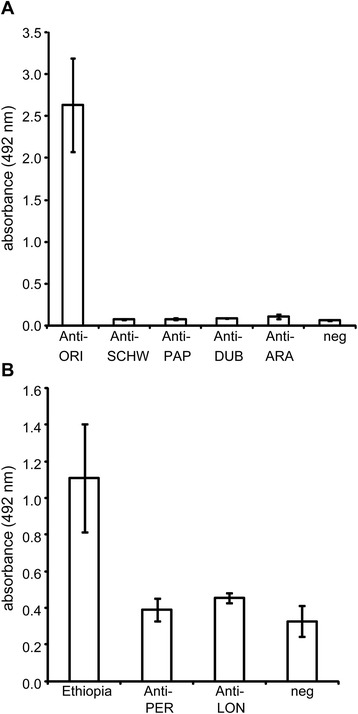
Specificity of the anti-sand fly saliva IgG antibody response. The reactivity of *Phlebotomus orientalis* salivary gland homogenates (SGH) with sera from mice (**a**) and dogs (**b**) repeatedly exposed to a single sand fly species was measured via ELISA. In graph A, SGH was incubated with sera from mice exposed to *P. orientalis* (Anti-ORI), *Sergentomyia schwetzi* (Anti-SCHW), *P. papatasi* (Anti-PAP), *P. duboscqi* (Anti-DUB), or *P. arabicus* (Anti-ARA). Each bar represents the mean for two serum samples ± the standard error. The values for the positive controls (the sera of mice incubated with homologous antigen) were as follows: *S. schwetzi* = 1.48 ± 0.43, *P. papatasi* = 2.38 ± 0.32, *P. duboscqi* = 2.87 ± 0.60, and *P. arabicus* = 1.86 ± 0.24. In graph B, the SGH was incubated with the sera from dogs exposed to *P. perniciosus* (Anti-PER) or *Lutzomyia longipalpis* (Anti-LON). Seropositive Ethiopian dogs (Ethiopia) and dogs that had never been exposed to sand flies (neg) were used as positive and negative controls, respectively. Each bar represents the mean of five serum samples ± the standard error. The absorbencies of the sera incubated with the homologous antigen were 2.42 ± 0.06 for *L. longipalpis* and 1.73 ± 0.13 for *P. perniciosus*

To verify the specificity of the anti-*P. orientalis* saliva antibodies we used sera from dogs and mice that had been experimentally exposed to a single sand fly species. In dogs, the reactivity of anti-*P. perniciosus* and anti-*Lutzomyia longipalpis* sera against *P. orientalis* salivary gland homogenate (SGH) was similar to that for sera from non-exposed dogs (Fig. 3a). However, all of the selected canine sera of Ethiopian origin reacted strongly to *P. orientalis* SGH (Fig. 3a). In mice, the *P. orientalis* salivary antigen reacted strongly only to the homologous IgGs (Fig. 3b). The reactivities of all heterologous antigen-antibody combinations were similar to those for sera from non-exposed mice (Fig. 3b).

### Correlation analysis of serological results

A positive correlation was found between the levels of anti-*P. orientalis* and anti-*L. donovani* IgG in Ethiopian cows (ρ = 0.37, p = 0.0001), goats (ρ = 0.37, p < 0.0001), and sheep (ρ = 0.65, p < 0.0001) (Table 5). This correlation remained significant even when the locality was considered, except for the cows from Humera, for which the correlation was only slightly outside of the level of significance (ρ = 0.48, p = 0.057). No significant correlation was found for the canine and donkey sera (Table 5).

**Table 5:** Correlation analysis of serological results

Species		Ethiopia	Addis Zemen	Sheraro	Humera
Cow	ρ	0.37***	0.38**	0.43*	0.48
	n	104	62	26	16
Dog	ρ	0.12	0.15	−0.46	0.36
	n	34	19	7	8
Donkey	ρ	0.31	0.50	0.52	−0.03
	n	20	3	11	6
Goat	ρ	0.37***		0.36***	0.37***
	n	239		106	133
Sheep	ρ	0.65***	0.67***	1.00***	0.61***
	n	149	27	5	117

Results from the Spearman-Rank Correlation Matrix test for anti-*Leishmania donovani* IgG and anti-*Phlebotomus orientalis* saliva IgG

*ρ* correlation coefficient, *n* number of serum samples tested

Asterisk (*) indicate significant correlations: *p < 0.05, **p < 0.01, ***p < 0.001

## Discussion

Visceral leishmaniasis is considered to be an anthroponosis in northwestern Ethiopia, but in nearby Sudanese foci, zoonotic transmission has also been suspected, with dogs and mongooses serving as possible reservoirs [3–5, 19]. With regard to domestic animals, sleeping near dogs, cattle, goats, or donkeys has been associated with an increased risk of VL in migrants and residents of Humera [20]. Understanding the mode of disease transmission, whether anthroponotic or zoonotic, is critical for the planning and implementation of effective VL control programs. Thus, one of the main goals of our study was to screen domestic animals for *Leishmania* DNA and discuss their possible involvement in the epidemiology of VL in Ethiopia as possible parasite hosts.

We evaluated two parameters associated with the ability of an animal to be a host for *Leishmania* parasites [21, 22]: (1) exposure to a sand fly vector as a source of blood and (2) the presence of *Leishmania* DNA in the animal’s peripheral blood.

In northwestern Ethiopia, the sand fly vector species of *L. donovani* has not yet been identified. However, *Phlebotomus orientalis* is the most probable vector given that it has been found to be infected with *L. donovani* in nearby Sudanese foci [7] and its susceptibility to this *Leishmania* species has been demonstrated experimentally [8]. Exposure to *P. orientalis* was assessed using anti-sand fly saliva antibodies as a marker [6]. Anti-saliva IgG antibodies were found in all of the animal species tested, which is indicative of the opportunistic feeding behavior of *P. orientalis* [23], thus meeting one criteria for the possible zoonotic transmission of *L. donovani*. Feeding preferences, together with other ecological constraints such as the localization of vector breeding sites [24] or vector susceptibility to harboring *Leishmania* infection [8] may help us to understand the complex picture of the ecology and transmission dynamics of VL in Ethiopia.

The presence of *Leishmania* DNA in animal peripheral blood and *Leishmania* seropositivity serve as reliable epidemiological markers for assessing infection. PCR positivity indicates the presence of the parasite [25, 26]. Although this technique cannot prove the intact integrity of the parasite, viability of the detected *Leishmania* is highly probable given that its DNA degrades shortly after parasite death [27]. Seropositivity, on the other hand, is considered a marker of exposure to *Leishmania* infection [28]. The majority of *Leishmania*-positive animals were found in Humera, indicating dynamic transmission to domestic animals in this well-known active focus. However, many *L. donovani*-seropositive animals were found in all the three surveyed localities, suggesting that exposure to *Leishmania* parasites also occurred in the foci of Addis Zemen and Sheraro.

The fact that only one-third of the PCR-positive animals were positive for both kDNA and ITS1-HRM PCR, is not surprising because the ITS1 region has a considerably lower copy number [11, 12]. Due to the small amount of parasite DNA available in blood samples, distinguishing between the closely related species *L. donovani* and *L. infantum* is notoriously difficult [15]. Moreover, distinction within the *L. donovani* complex in East Africa is controversial; strains that were previously split into *L. donovani*, *L. archibaldi* or *L. infantum* have now been classified into one group: *L. donovani* s.s. [29].

The most suspected animal reservoirs for *L. donovani* are dogs, which are known to play a key role as reservoir hosts in the transmission cycle of the closely related *L. infantum* [2, 30]. Several authors have reported PCR-positivity or seropositivity of dogs in *L. donovani* foci [3, 4, 19, 31–35], including Humera and Addis Zemen in Ethiopia [36–39]. In the present study, dogs demonstrated the highest *Leishmania* seroprevalence out of all the species tested at all study sites, with two PCR-positive dogs identified in Humera and Addis Zemen. As a suspected reservoir species, dogs are also highly attractive to the vector [35], which is supported by our findings that dogs exhibited the highest seroprevalence of anti-*P. orientalis* antibodies among the tested animal species. Most importantly, the same *Leishmania* strains have been recovered from dogs and VL patients [3, 4, 19] and have been shown to persist in dogs for years [19]. Dogs have been recognized as a risk factor for human VL [20, 37, 39], and as the most probable reservoir hosts, their involvement in disease transmission should be addressed in control strategies for VL caused by *L. donovani*.

Almost 38 % of *Leishmania*-positive animals have also been found to be seropositive, indicating these domestic animals (donkeys, goats, sheep) as putative host species in local VL foci. Nevertheless, it is important to mention that neither PCR-positivity nor seropositivity indicates that an animal is able to maintain the parasite for a long period of time. This must be primarily demonstrated by the follow-up of infected animals. Several studies of naturally or experimentally infected non-canine domestic animals have demonstrated their different capabilities to maintain *Leishmania* infection. Cerqueira *et al*. [40] experimentally infected four donkeys with *L. chagasi* (syn. *L. infantum*). These donkeys remained seropositive until the end of the study, which lasted 12 months; however, the donkeys were able to overcome the infection and failed to infect the vector [40]. A PCR survey reported by Bhattarai *et al*. indicated that *Leishmania* infection in goats can persist for at least seven months [41]. On the other hand, *L. donovani* infection in sheep is likely time-limited because only one out of six experimentally infected sheep was shown to develop measurable amounts of anti-*L. donovani* antibodies and the transient presence of amastigotes in sampled tissue in a study that included 244 days of monitoring [42]. Thus, the 37.6 % seropositivity detected in our study may indicate a high infection rate among Ethiopian sheep, further supported by the significantly higher levels of anti-*L. donovani* IgG antibodies among *Leishmania*-positive sheep (Additional files 3 and 4).

The fact that many animals were seropositive for *Leishmania* while PCR-negative in the blood, and, on the other hand, that out of 32 PCR-positive animals, 20 animals were seronegative, could be explained by several possible mechanisms. Seropositivity and PCR-negativity might be attributable to infection in hosts that have resolved the infection but retain high titers of specific antibodies [40, 43]. Another possibility is that seropositive animals might carry the infection in their tissues without parasitemia and are therefore negative according to blood PCR [44]. The reverse situation with PCR-positivity and seronegativity could be attributable to the delayed development of a detectable antibody response in early infection [45], or due to an infection in animals whose B-cells are unresponsive to *Leishmania* antigens, as found in some asymptomatic hosts [2, 43, 44].

The role of other domestic animals as hosts or potential reservoirs for *L. donovani* is still unclear. The present study is the first to report PCR-positive cattle, donkeys, goats, and sheep in Ethiopia. These animals, especially cattle, serve as sources of blood for *L. donovani* vectors [23, 46]. Even if these species do not serve as reservoir hosts for the parasite, they still attract large numbers of blood-questing female sand flies and may, therefore, act as a protective barrier in the case of resistant or refractory mammal species or as a risk factor in the case of susceptible species [30, 37, 47]. Prediction of the role of domestic animals in the amplification or dilution of VL risk might be possible using a recently described mathematical model for multi-host infectious diseases by applying relevant data [48].

In addition to the maintenance of persistent infection, the transmissibility competence, e.g. infectivity for the sand fly vector, is an important prerequisite for any mammal to serve as a *Leishmania* reservoir [28, 49]. These two criteria, among other aspects, can distinguish between a reservoir host and an incidental host that is not capable of infecting the vector [25]. Validation of these prerequisites for domestic animals in northwest Ethiopia, however, requires further investigation.

## Conclusions

In conclusion, leishmaniasis caused by *L. donovani* is traditionally considered to be an anthroponosis in East Africa. However, the present study revealed widespread exposure to *L. donovani* and sand fly vector bites among domestic animals. The possible involvement of domestic animals as sources of blood for vector sand flies should therefore be considered in VL control strategies. However, the direct involvement of domestic animals in the transmission cycle of *L. donovani* warrants further investigation, most importantly by xenodiagnosis to determine their transmissibility competence.

## Additional files

Additional file 1:
**Accession numbers for** ***Leishmania*** **ITS sequences downloaded from the GenBank database and used for the phylogenetic analysis presented in Fig.** 1 **.**
Additional file 2:
**Details of the ELISA methods.**
Additional file 3:
**Detailed list of Ethiopian animals positive for** ***Leishmania*** **DNA.**
Additional file 4:
**Differences in the levels of anti-** ***Leishmania donovani*** **IgG and anti-** ***Phlebotomus orientalis*** **saliva IgG between** ***Leishmania*** **-positive (full circle) and** ***Leishmania*** **-negative (open circle) animals in the Humera region (the majority of PCR-positive animals are from this locality: 30 out of 32).** Significant differences are marked by the probability level on the X-axis.

## Abbreviations

ELISA: Enzyme-linked immunosorbent assay
ITS1: Internal transcribed spacer 1
kDNA: Kinetoplast deoxyribonucleic acid
*L.*: *Leishmania* or *Lutzomyia*
OD: Optical density
*P.*: *Phlebotomus*
PBS: Phosphate-buffered saline
PBS-Tw: Phosphate-buffered saline with Tween
PCR: Polymerase chain reaction
SGH: Salivary gland homogenate
VL: Visceral leishmaniasis
