# Supplementary material for: Exposure to Leishmania spp. and sand flies in domestic animals in northwestern Ethiopia
Source: Parasit Vectors. 2015 Jul 8;8:360. doi: 10.1186/s13071-015-0976-1 (PMC4495613; doi:10.1186/s13071-015-0976-1)
Supplement: Additional file 1: — Accession numbers for Leishmania ITS sequences downloaded from the GenBank database and used for the phylogenetic analysis presented in Fig. 1 . [file 13071_2015_976_MOESM1_ESM.doc]

**Additional file 1 - Accession numbers for *Leishmania* ITS sequences downloaded from the GenBank database and used for the phylogenetic analysis presented in Figure 1.**

*Leishmania donovani:*

AB725909, AJ000292, AJ000291, AJ000293, AJ000294, AJ000295, AJ000296, AJ000297, AJ249611, AJ249612, AJ249613, AJ249614, AJ249615, AJ249620, AJ249621, AJ249622, AJ276259, AJ276260, AJ634356, AJ634361, AJ634374, AJ634375, AJ634376, AJ634377, AM901448, AM901449, AM901450, AM901451, AM901452, EU326227, FJ753386, FN182206, FN182208, FN182209, FN182210, FN398344, FN677358, FN677359, FN677363, FN677364, GU045588, GU045589, GU045591, HG512907, HG512911, HG512916, HG512920, HG512926, HG512936, HG512944, HG512953, HM130608, HQ830354, JN181861, JN181862, JQ730001, JQ730002, KF500031, KF525783, KF543268, KF543269, KF543270, KF673344, KF673345

*Leishmania infantum:*

AJ000288, AJ000289, AJ000295, AJ000303, AJ000304, AJ000305, AJ000306, AJ634339, AJ634340, AJ634355, AJ634361, AJ634362, AJ634370, AJ634371, EU326228, EU604810, FJ497004, FJ555210, FM164418, FM164419, FM164420, FN398341, FN398343, GQ367486, GQ367487, GU045591, GU045592, HG512913, HG512917, HG512921, HG512928, HG512941, HG512943, HG512947, HG512955, HG512956, HQ535858, JX289852, JX289853, JX289879, JX289880, JX448536, JX448537, JX448538, JX448539, JX448541, JX448542, JX448543, JX448545, JX945644, KC347299, KC347300, KC347301, KC355188, KC477100, KC570454, KC686340, KC686341, KC998879, KF705513, KF705514, KF705515

A sample from Humera, Ethiopia:

KJ010540
