# Supplementary material for: Exposure to Leishmania spp. and sand flies in domestic animals in northwestern Ethiopia
Source: Parasit Vectors. 2015 Jul 8;8:360. doi: 10.1186/s13071-015-0976-1 (PMC4495613; doi:10.1186/s13071-015-0976-1)
Supplement: Additional file 2: — Details of the ELISA methods. [file 13071_2015_976_MOESM2_ESM.doc]

**Additional file 2 - Details of the ELISA methods**

|  | **Cow** | **Dog** | **Donkey** | **Goat** | **Sheep** |
| --- | --- | --- | --- | --- | --- |
| **Blocking solution and dilutant for serum samples** | normal chicken serum, Vector, S-3000 | low fat dry milk, Bio-Rad, 170-6404 | normal rabbit serum, Vector, S-5000 | normal rabbit serum, Vector, S-5000 | inactivated horse serum, Gibco, 16050-122 |
| **Sample dilution**  **for anti-*Leishmania* IgG** | 1:200 | 1:50 | 1:50 | 1:50 | 1:50 |
| **Sample dilution for anti-saliva IgG** | 1:200 | 1:200 | 1:50 | 1:50 | 1:50 |
| **Conjugate** | Goat anti-bovine IgG, Jackson Immuno Research, 101-035-003 | Sheep anti-dog IgG, Bethyl Laboratories, A40-118P | Rabbit anti-donkey IgG, Novus Biological, NB120-6765 | Rabbit anti-goat IgG, Novus Biological, NB710-H | Rabbit anti-sheep IgG, Novus Biological, NB7195 |
| **Conjugate dilution** | 1:10.000 | 1:3.000 | 1:5.000 | 1:5.000 | 1:10.000 |
