# Supplementary material for: Exposure to Leishmania spp. and sand flies in domestic animals in northwestern Ethiopia
Source: Parasit Vectors. 2015 Jul 8;8:360. doi: 10.1186/s13071-015-0976-1 (PMC4495613; doi:10.1186/s13071-015-0976-1)
Supplement: Additional file 3: — Detailed list of Ethiopian animals positive for Leishmania DNA. [file 13071_2015_976_MOESM3_ESM.doc]

**Additional file 3 – Detailed list of Ethiopian animals positive for *Leishmania* DNA.**

Three cows, 2 dogs, 2 donkeys, 16 goats, and 9 sheep were found to be PCR-positive for *Leishmania* DNA. The table provides information about the animal identification number (ID), its geographical origin (Locality), positivity (+) or negativity (-) for *Leishmania* DNA in both PCR methods (ITS1 HRM and kDNA), and the levels of anti-*L. donovani* and anti-*P. orientalis* IgG antibodies (Anti-Leish IgG and Anti-Ori IgG, respectively). An asterisk (*) indicates animal seropositivity based on the cut-off values listed in Table 3 and Table 4.

| **Animal ID** | **Species** | **Locality** | ***Leishmania***  **ITS1- HRM**  **PCR** | ***Leishmania* kDNA PCR** | **Anti-Leish IgG** | **Anti-Ori IgG** |
| --- | --- | --- | --- | --- | --- | --- |
| 1 | cow | Humera | + | + | 0.566 | 0.320 |
| 2 | cow | Humera | - | + | 1.058 | 0.438 |
| 3 | cow | Humera | - | + | 0.560 | 0.334 |
| 4 | dog | Addis Zemen | + | + | 0.195 | 0.702* |
| 5 | dog | Humera | - | + | 0.168 | 0.171* |
| 6 | donkey | Sheraro | - | + | 0.754* | 0.609* |
| 7 | donkey | Humera | - | + | 0.403 | 0.437 |
| 8 | goat | Humera | + | + | 0.247 | 0.532 |
| 9 | goat | Humera | + | + | 0.416 | 0.593 |
| 10 | goat | Humera | + | + | 0.484 | 0.525 |
| 11 | goat | Humera | - | + | 1.343* | 0.412 |
| 12 | goat | Humera | - | + | 1.065* | 0.491 |
| 13 | goat | Humera | - | + | 0.804* | 0.489 |
| 14 | goat | Humera | - | + | 0.631 | 0.482 |
| 15 | goat | Humera | - | + | 0.526 | 0.280 |
| 16 | goat | Humera | - | + | 0.494 | 0.619 |
| 17 | goat | Humera | - | + | 0.482 | 0.264 |
| 18 | goat | Humera | - | + | 0.443 | 0.639 |
| 19 | goat | Humera | - | + | 0.349 | 0.423 |
| 20 | goat | Humera | - | + | 0.282 | 0.408 |
| 21 | goat | Humera | - | + | 0.251 | 0.396 |
| 22 | goat | Humera | - | + | 0.192 | 0.376 |
| 23 | goat | Humera | - | + | 0.187 | 0.346 |
| 24 | sheep | Humera | + | + | 0.788* | 0.556* |
| 25 | sheep | Humera | + | + | 0.766* | 0.326 |
| 26 | sheep | Humera | + | + | 0.720* | 0.491* |
| 27 | sheep | Humera | + | + | 0.712* | 0.403 |
| 28 | sheep | Humera | - | + | 1.074* | 0.959* |
| 29 | sheep | Humera | - | + | 1.003* | 0.537* |
| 30 | sheep | Humera | - | + | 0.930* | 0.804* |
| 31 | sheep | Humera | - | + | 0.728* | 0.405 |
| 32 | sheep | Humera | - | + | 0.433 | 0.270 |
|  |  |  |  |  |  |  |
