# Supplementary material for: Exposure to Leishmania spp. and sand flies in domestic animals in northwestern Ethiopia
Source: Parasit Vectors. 2015 Jul 8;8:360. doi: 10.1186/s13071-015-0976-1 (PMC4495613; doi:10.1186/s13071-015-0976-1)
Supplement: Additional file 4: — Differences in the levels of anti- Leishmania donovani IgG and anti- Phlebotomus orientalis saliva IgG between Leishmania -positive (full circle) and Leishmania -negative (open circle) animals in the Humera region (the majority of PCR-positive animals are from this locality: 30 out of 32). Significant differences are marked by the probability level on the X-axis. [file 13071_2015_976_MOESM4_ESM.doc]

**Additional file 4 –** Differences in the levels of anti-*Leishmania* *donovani* IgG and anti-*Phlebotomus* *orientalis* saliva IgG between *Leishmania*-positive (full circle) and *Leishmania*-negative (open circle) animals in the Humera region (the majority of PCR-positive animals are from this locality: 30 out of 32). Significant differences are marked by the probability level on the X-axis.

**
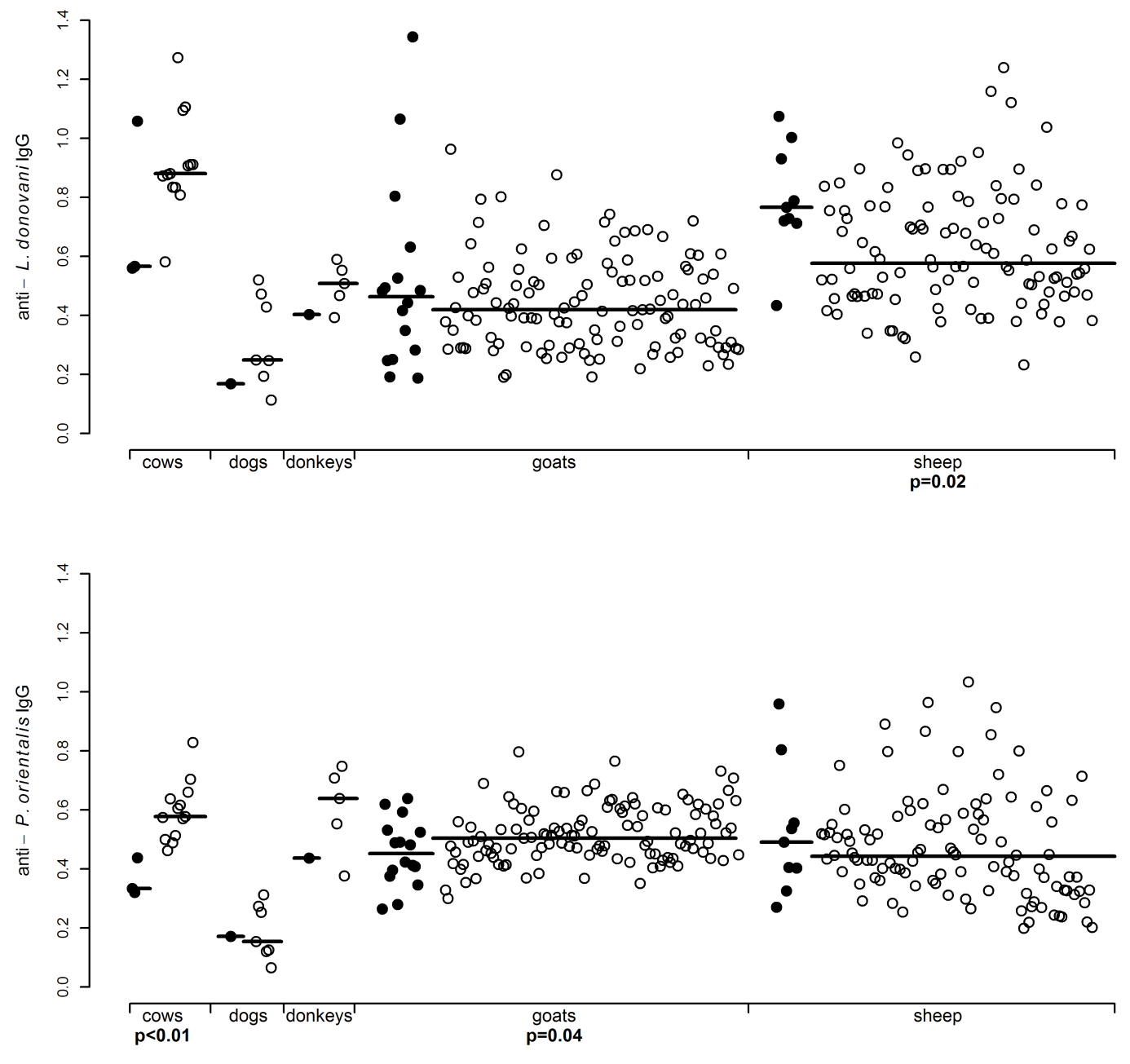
**
